# DNA methylation associated with the serum alanine aminotransferase concentration: evidence from Chinese monozygotic twins

**DOI:** 10.1186/s13148-025-01869-1

**Published:** 2025-04-28

**Authors:** Jingxian Li, Jia Luo, Tong Wang, Xiaocao Tian, Chunsheng Xu, Weijing Wang, Dongfeng Zhang

**Affiliations:** 1 Department of Epidemiology and Health Statistics, The School of Public Health of Qingdao University, No.308 Ningxia Road, Qingdao, 266071 Shandong Province People’s Republic of China; 2 Qingdao Municipal Centre for Disease Control and Prevention, No.175 Shandong Road, Qingdao, 266033 Shandong Province People’s Republic of China

**Keywords:** Alanine aminotransferase, Causality, DNA methylation, Monozygotic twins

## Abstract

**Background:**

To identify nongenetic factors influences on DNA methylation (DNAm) variations associated with blood Alanine Aminotransferase (ALT) concentration, this study conducted an epigenome-wide association study (EWAS) on Chinese monozygotic twins.

**Methods:**

A total of 61 pairs of Chinese monozygotic twins involved in this study. Whole blood samples were analyzed for DNAm profiling using the Reduced Representation Bisulfite Sequencing (RRBS) technique. We examined the relationship between DNAm levels at each CpG site and serum ALT using a linear mixed-effects model. Enrichment analysis and causal inference analysis was conducted, and differentially methylated regions (DMRs) were further identified. Candidate CpGs were validated in a community sample. Genome-wide significance were calculated by Bonferroni correction (*p* < 2.14 × 10^–7^).

**Results:**

We identified 85 CpGs reaching genome-wide significance (*p* < 2.14 × 10^–7^), located in 16 genes including *FLT4*, *ADARB2*, *MRPS31P2*, and *RELB*. Causal inference suggested that DNAm at 61 out of 85 significant CpGs within 14 genes influenced ALT level. 52 DMRs and 1765 pathways such as low voltage-gated calcium channel activity and focal adhesion were identified having influences on ALT levels. Further validation using community population found four CpGs mapped to *FLT4* and three to *RELB* showing hypomethylation and hypermethylation in cases with abnormal ALT (ALT > 40 U/L), respectively.

**Conclusion:**

This study identified several differentially methylated CpG sites associated with serum ALT in the Chinese population, particularly within *FLT4* and *RELB*. These findings provide new insights into the epigenetic modifications underlying liver function.

**Supplementary Information:**

The online version contains supplementary material available at 10.1186/s13148-025-01869-1.

## Background

Alanine aminotransferase (ALT) is a critical enzyme that is predominantly located in the liver, and its elevated levels are a frequent indicator of liver damage [1, 2]. In addition to indicating liver damage, elevated ALT levels also function as a predictive marker for a variety of ailments and all-cause mortality [3–5]. Consequently, it is imperative to comprehend the factors that influence the serum ALT level in order to promote human health.

As a complex trait, serum ALT concentration may be influenced by a combination of genetic and environmental factors. The extent of genetic contributions to ALT variability has been widely studied, revealing significant correlations between genetic factors and ALT levels. Previous studies found that ALT concentrations exhibit high heritability although in different races, ranging from 22 to 65% [6–11]. Moreover, the genetic pathways underlying variations in ALT levels have also begun to be uncovered by genome-wide association studies, or genome-wide association studies (GWASs). Investigations in European whites, Indian Asians [12], Mexican Americans [13], and the Japanese population [14] have pinpointed various loci and genes, including *PNPLA3* and *SAMM50*, associated with ALT levels. Nonetheless, the previously identified genetic variants account for only a part of the genetic basis of ALT.

Recent years have provided compelling evidence of the crucial role that epigenetic mechanisms play in altering gene expression and increasing disease risk. Several epigenome-wide association studies (EWASs) have been carried out to investigate the relationship between complicated traits such as blood pressure [15], blood lipids [16], body mass index (BMI) and waist circumference [17], type 2 diabetes [18, 19], cardiovascular disease [20] and genomic DNA methylation (DNAm) variations. However, while some studies have explored the correlation between DNAm and ALT [21], studies specifically focused on East Asian populations are particularly limited. This gap is particularly important because previous studies have demonstrated differences in DNA methylation across ethnicities/races, suggesting that ethnicity/race may be an influential factor in epigenetic regulation [22–24]. Moreover, the causal relationship—whether DNAm directly affects serum ALT levels—remains unclear. Therefore, further EWAS and causal inference analyses are essential.

Both genetic and non-genetic factors can contribute to epigenetic modifications [25]. However, previous EWASs primarily utilized samples from unrelated individuals, which often overlooked confounding effects arising from diverse genetic backgrounds. Fortunately, this limitation can be addressed using monozygotic twin models, as those twins share identical genetic information. This design effectively mitigates biases associated with individual genetic variations, thereby providing a clearer understanding of the relationship between DNAm caused by external factors (such as diet, smoking, and alcohol consumption) and disease risk [26, 27].

In this study, we leveraged an ALT-discordant monozygotic twin design, where co-twins within each pair exhibited different serum ALT levels, to conduct an EWAS exploring the correlation between DNAm at specific CpG sites and ALT levels. This discordant twin approach is particularly advantageous, as it controls for shared genetics and many early environmental influences, allowing for more precise identification of epigenetic variations associated with ALT. Furthermore, we explored the potential causal relationship between DNAm and ALT levels. To validate our findings, the identified candidate CpGs were subsequently tested in a community-based population sample.

## Materials and methods

### Participants

Participants were recruited through the Qingdao Twin Registry [28], with recruitment details previously documented [29]. Exclusions comprised individuals with hepatitis, pregnant or breastfeeding women, and those who were unconscious or unwilling to participate. A discordant trait monozygotic twin design was employed, selecting pairs with a difference in serum ALT levels of ≥ 1 U/L. Methylation analysis was conducted on 61 pairs of monozygotic twins (122 individuals) with discordant ALT levels, where the absolute median difference in serum ALT levels within pairs was 7.00 U/L (interquartile range [IQR]: 3.00–11.00). The median age of the participants was 52 years (IQR: 46–57), and 49.2% (n = 60) were women. These demographic characteristics of the participants are summarized in Table 1.

**Table 1:** Demographic and health information of monozygotic twins

n	ALT(U/L)	Age (years)	Gender woman (n, %)	BMI (kg/ m^2^,IQR)	Alcohol consumption (100 g/day, IQR)	Hypertension (n, %)	Diabetes (n, %)
122	20 (12,26)	52 (46,57)	60(49.2%)	25 (22.40,27.48)	0 (0,2)	67(54.92%)	12(9.84%)

^IQR: interquartile ranges^

The study was approved by the Regional Ethics Committee of the Institutional Review Board of the Qingdao Center for Disease Control and Prevention, adhering to the ethical principles of the Declaration of Helsinki. All participants provided written informed consent prior to the study.

### Data collection, health examination and zygosity determination

A health examination and a questionnaire were completed by each pair of cotwins. For the blood sample collection, each participant provided 10 millilitres of venous blood following an overnight 10–12 h fast. Serum and plasma were separated from blood cells and stored at − 80 °C for 30 min. A Hitachi 7600 semiautomatic analyzer from Japan was used to measure the concentrations of serum ALT and blood glucose [15, 16, 30].

### Covariates and personal information

The covariates in the current study are age, gender, alcohol consumption, body mass index, hypertension status, and diabetes status, as determined by previous research. A summary of these covariates is provided in Table 1.

Questionnaires were distributed at the local Qingdao CDC service center or at community hospitals/clinics to gather personal information, including age, sex, and alcohol consumption (as measured by the question "Currently, what is your average daily alcohol consumption?"). The formula weight (kg)/height (m)^2 was employed to determine BMI from the measured height and weight data. Hypertension was defined as a measured systolic blood pressure exceeding 140 mmHg, a diastolic blood pressure exceeding 90 mmHg, or self-reported hypertension. Self-report or fasting blood glucose levels exceeding 7 mmol/L were used to ascertain diabetes status.

### Reduced representation bisulfite sequencing (RRBS) experiment

Total DNA was isolated from the whole blood sample in the RRBS experiment. In brief, genomic DNA was digested using a restriction enzyme to generating short fragments with CpG-rich regions. Subsequently, bisulfite conversion was performed to analyze DNA methylation patterns. Raw methylation data spanning 551,447 CpGs per participant's genome was subsequently obtained by constructing and sequencing cDNA libraries. *Bismark* [31] was employed to align the methylation data to the human GRCh37 reference genome, and *BiSeq* [32] was employed to normalize the data, with coverage limited to the 90th percentile. The quality control process excluded CpGs with a mean methylation *β*-value < 0.01 or > 0.99 or more than 10 missing observations, resulting in 233,720 CpGs that were relevant to ALT for further analysis. Methylation *β*-value was log2-transformed into *M*-value for statistical analysis. Given that DNA was sourced from whole blood, accounting for cell type heterogeneity was crucial to avoid potential biases [33]. To mitigate this, we employed the *ReFACTor* method, utilizing the top five components to adjust for cell type composition effects on DNAm [34].

### Epigenome‑wide association analysis (EWAS)

Through the utilization of the *lmer* function in the R-package *lmerTest*, the linear mixed-effects model was implemented to assess the correlation between DNAm M-values (log2-transformed methylation β-values) at each CpG site and ALT levels. In this model, M-values were included as fixed-effect independent variables, while twin pair IDs were incorporated as a random effect to account for the paired structure of the twin data. This model adjusted for covariates including age, gender, alcohol consumption, BMI, hypertension status, diabetes status, and cell type composition. To account for the paired structure of twin data, a random effect was included to identify twin pairs within the linear mixing model. Bonferroni correction was applied to adjust for multiple testing of 233,720 CpGs, setting a genome-wide significance threshold at *p* < 2.14 × 10^–7^ (Bonferroni correction *p* value < 0.05) [35]. The R package *biomaRt* was used to annotate CpGs with *p* < 0.05 to the nearest gene [36, 37].

### Power estimation for EWAS

Given the lack of a standardized sample size formula for discordant monozygotic twin designs, we estimated statistical power through simulation-based approaches, which evaluated the statistical power of EWAS using a disease-discordant twin design [26]. The study demonstrated that both higher phenotypic heritability (h2) and a stronger correlation between DNA methylation and environmental factors (R2_M,E_) lead to a smaller required sample size to achieve adequate power, assuming other factors remain constant.

To adopt a conservative approach, we selected R2_M,E_ = 0.1, as a lower DNA methylation–environment correlation results in a larger estimated sample size. For h2, while a lower value would be more conservative, we set h2 = 0.6, which is slightly lower than the previously reported ALT heritability (h2 = 0.65) in the Chinese population [11]. This choice ensured that our power estimation remained biologically plausible while accounting for potential variability.

Under an extreme case where the intra-pair correlation due to shared genetics or environment *ρε* = 0.1, the simulation results indicated that a sample size of 63 pairs would be sufficient to achieve 80% power. This suggests that a sample size of approximately 60 twin pairs is adequate for reaching the desired statistical power in disease-discordant twin EWAS studies. Therefore, we estimated that our study, which included 61 twin pairs, will achieve approximately 80% statistical power.

### Causal inference analysis

To assess the potential causal relationship between serum ALT levels and CpGs identified at the epigenome-wide significance level (*p* < 2.14 × 10⁻⁷), we applied the Inference about Causation through Examination of Familial Confounding (ICE FALCON) method. ICE FALCON is a regression-based approach specifically designed for causal inference in family-based studies, particularly in twin studies, as it accounts for both genetic and shared environmental confounders [38, 39].

Based on this method, we fitted three regression models to the twin-pair data:

Model 1: The outcome variable (serum ALT level) of each twin was regressed on their own predictor variable (CpG methylation) to estimate the unconditional regression coefficient “*β*_self_”, which captures both the causal effect and familial confounding.

Model 2: The outcome variable of each twin was regressed on their co-twin’s predictor variable to estimate “*β*_co-twin_”, which reflects the influence of familial confounding alone.

**Fig. 1:**
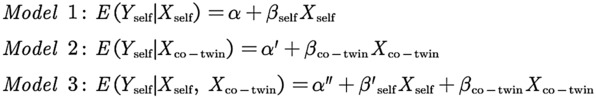
Manhattan plot of the effects of EWASs on ALT levels

Model 3: The outcome variable was regressed on both the twin’s own predictor and their co-twin’s predictor to obtain the conditional regression coefficients “*β’*_self_” and “*β’*_co-twin_” Unlike “*β*_self_” and “*β*_co-twin_”, these coefficients indicate the change in outcome associated with a change in the predictor while keeping the other predictor constant, allowing a more refined causal interpretation. The regression models for the ICE FALCON method are presented in Fig. 1.

The criteria for causal inference were as follows:

If the absolute difference between |*β*_co-twin_—*β’*_co-twin_| is similar to |*β*_self_—*β’*_self_|, then the observed association is likely due to familial confounding rather than causality.

Conversely, a causal effect is indicated if the absolute value of the ratio: |*β*_co-twin_—*β’*_co-twin_|/ |*β*_self_—*β’*_self_| (absolute value of ratio) is greater than 1.5, suggesting that within-pair differences in CpG methylation correspond to within-pair differences in ALT levels, independent of familial confounding.

To estimate the parameters, we employed a generalized estimating equations model, which accounts for the correlation structure within twin pairs. The rationale behind these statistical criteria has been supported by previous methodological studies [38, 40].

### Region‑based analysis

The *comb-p* was implemented to identify differentially methylated regions (DMRs) that are linked to ALT [41]. A region was considered significantly enriched with DMRs if the Stouffer-Liptak-Kechris (*slk*) corrected p-value was less than 0.05.

### Ontology enrichments analysis

The ontology enrichment of identified CpGs (*p* < 0.05) was evaluated using the online instrument Genomic Regions Enrichment of Annotations instrument (GREAT) [42].

Annotations were conducted using the human GRCh37 genome reference and the default "basal plus extension" association rule. An ontology was considered statistically significant if its Bonferroni correction *p* value was less than 0.05.

### Validation using independent sample

We selected genes for quantitative methylation analysis to validate our EWAS results based on following criteria: 1) results of top signals in EWAS associated with our target trait; 2) the gene was involved in important biological function and pathways that may potentially influence; 3) the gene was where the CpGs having causal relationship with ALT in the causal inference analysis were located; 4) the primers of the gene could be successfully designed.

Blood collection and preservation procedures were consistent with previous methods. Validation of DNAm for the top CpGs was performed using matrix-assisted laser desorption ionization time-of-flight (MALDI-TOF) mass spectrometry. Mass spectra were obtained using a MassARRAY Compact MALDI-TOF system (Sequenom; BioMiao Biological Technology, Beijing, China), and methylation ratios were generated with the EpiTYPER software (Sequenom, San Diego, CA). Multiple primer schemes were designed to cover regions containing the top CpGs, with final primer optimization carried out using Epidesigner software. The EpiTYPER™ software system facilitated quantitative DNAm analysis, and the mean methylation status of closely adjacent CpGs was computed.

To validate our EWAS results, we conducted an independent replication analysis in a community-based cohort from Qingdao, China. Participants were included if they were aged ≥ 40 years and had available data on ALT levels, BMI, alcohol intake, diabetes status, and blood pressure. Individuals with active hepatitis and pregnant or breastfeeding women, were excluded from this cohort. The validation cohort consisted of 54 individuals with abnormal ALT levels (ALT > 40 U/L) and 162 matched controls, selected using propensity score matching (PSM) to minimize potential confounding effects. Cases and controls were matched at a 1:3 ratio based on age and sex, using nearest-neighbor PSM with a caliper value of 0.1. This method ensures that the matched individuals are comparable in baseline characteristics, improving the validity of our findings. The effect of DNAm to ALT was evaluated using the conditional logistic regression model, which was adjusted for match group, BMI, alcohol intake, diabetes, and hypertension status.

## Results

### Epigenome-wide association analysis

Sixty-one pairs of monozygotic twins were included in the methylation study; their median serum ALT value was 20 U/L (IQR: 12–26). The demographic and clinical characteristics of the participants are summarized in Table 1. The median age was 52 years (IQR: 46–57), and 49.2% (n = 60) were women. The median ALT level was 20 U/L (IQR: 12–26), and the median BMI was 25.0 kg/m^2^ (IQR: 22.40–27.48). Among the participants, 54.92% (n = 67) had hypertension, and 9.84% (n = 12) had diabetes. The median alcohol consumption was 0 g/day (IQR: 0–2).

**Fig. 2:**
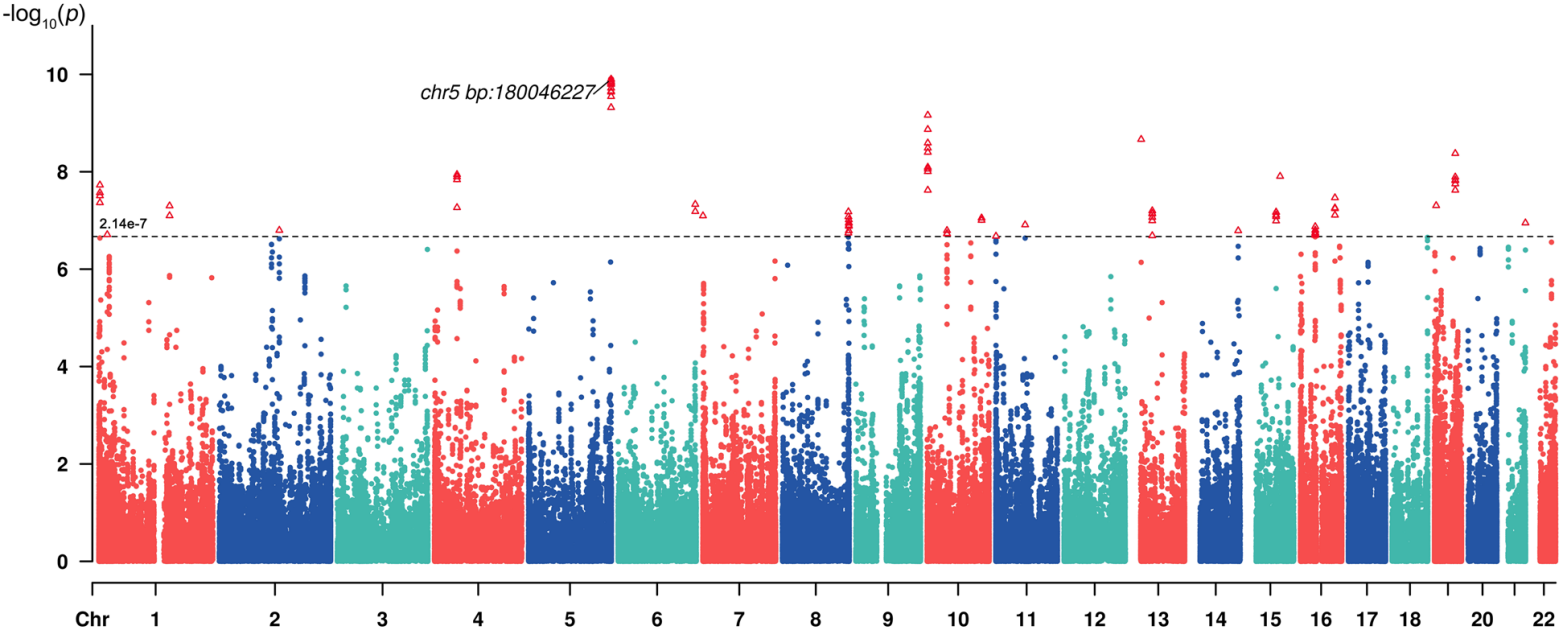
Differentially Methylated Regions (DMRs) associated with serum ALT Levels

Figure 2 displays the Manhattan plot for the EWAS on ALT levels. We identified 85 ALT-related CpG sites with *p* values less than 2.14 × 10^–7^. Table 2 displays the top 35 significant CpG sites, while all 85 significant CpG sites are detailed in Table S1.

**Table 2:** Top 35 ^a^Significant CpG Sites Associated with ALT Levels

Chromosome	Position	*p* value	*β*	Gene	Gene function^b^
chr5	180,046,227	1.26E-10	− 0.089	*FLT4*	A tyrosine kinase receptor involved in lymphangiogenesis and endothelial maintenance
chr5	180,046,224	1.26E-10	− 0.089	*FLT4*	
chr5	180,046,235	1.38E-10	− 0.089	*FLT4*	
chr5	180,046,240	1.47E-10	− 0.089	*FLT4*	
chr5	180,046,244	1.53E-10	− 0.089	*FLT4*	
chr5	180,046,249	1.60E-10	− 0.089	*FLT4*	
chr5	180,046,258	1.91E-10	− 0.088	*FLT4*	
chr5	180,046,270	2.22E-10	− 0.088	*FLT4*	
chr5	180,046,275	2.30E-10	− 0.088	*FLT4*	
chr5	180,046,290	2.85E-10	− 0.089	*FLT4*	
chr5	180,046,305	4.79E-10	− 0.089	*FLT4*	
chr10	1,517,277	6.88E-10	− 0.084	*ADARB2*	An RNA-editing enzyme involved in RNA regulation
chr10	1,517,258	1.35E-09	− 0.086	*ADARB2*	
chr13	20,139,121	2.16E-09	− 0.070	*MRPS31P2*	–
chr10	1,517,242	2.56E-09	− 0.087	*ADARB2*	An RNA-editing enzyme involved in RNA regulation
chr10	1,517,237	3.26E-09	− 0.088	*ADARB2*	
chr10	1,517,307	3.98E-09	− 0.082	*ADARB2*	
chr19	45,532,179	4.21E-09	0.066	*RELB*	A transcription factor involved in immune regulation and lymphocyte differentiation
chr10	1,517,310	8.07E-09	− 0.081	*ADARB2*	
chr10	1,517,225	8.79E-09	− 0.089	*ADARB2*	
chr10	1,517,219	9.89E-09	− 0.089	*ADARB2*	
chr4	49,163,502	1.14E-08	0.063	–	
chr15	72,490,479	1.24E-08	0.067	–	
chr4	49,163,509	1.27E-08	0.063	–	
chr19	45,532,170	1.28E-08	0.064	*RELB*	
chr4	49,163,519	1.45E-08	0.063	–	
chr19	45,532,208	1.47E-08	0.080	*RELB*	
chr19	45,532,201	1.52E-08	0.079	*RELB*	
chr19	45,532,214	1.52E-08	0.079	*RELB*	
chr19	45,532,196	1.77E-08	0.079	*RELB*	
chr1	2,928,715	1.87E-08	0.066	–	
chr19	45,532,235	2.39E-08	0.080	*RELB*	
chr10	1,517,313	2.39E-08	− 0.081	*ADARB2*	
chr1	2,928,748	2.69E-08	0.066	–	
chr1	2,928,733	3.11E-08	0.066	–	

^a^The 35 CpGs represent the top significant sites based on the linear mixed-effects model results, with the full list available in the Table S1. ^b^Gene function information from the National Center for Biotechnology Information (NCBI) https://www.ncbi.nlm.nih.gov/

The 11 strongest associations (*β* = − 0.089 to − 0.088, *p* = 1.26 × 10^–10^ to 4.79 × 10^–10^) were found for the CpG sites located on chromosome 5 (180,046,224 to 180,046,305 bp) within the FLT4 gene, all of which presented significant statistical associations (*p* < 2.14 × 10^–7^).

These top CpG sites were located within 16 genes, including *FLT4*, *ADARB2*, *MRPS31P2*, *RELB*, *DACT2*, *MATK*, *ZBTB7B*, *ENSG00000259268*, *ENSG00000240093*, *VAX1*, *OPLAH*, *PCNT*, *PLCB3*, *WDR20*, *LY6E*, and *PAX7*. These genes were also found near 10 additional genes (refer to Tables 2 and S1).

### Causal inference analysis

Table 3 displays the causal inference analysis results for the top 35 CpGs. The comprehensive results for the top CpGs (n = 85, *p* < 2.14 × 10^–7^) are detailed in Table S2. The findings strongly support a causal effect of DNAm on ALT levels for 61 CpGs located within 14 genes: *FLT4*, *MRPS31P2*, *ADARB2*, *RELB*, *DACT2*, *MATK*, *ZBTB7B*, *ENSG00000259268*, *VAX1*, *OPLAH*, *PCNT*, *PLCB3*, *LY6E*, *PAX7,* and nearly 7 other genes. Notably, among these 61 significant CpGs, only one CpG located in *PCNT* (chr21, position: 47,808,888), which is located in the *PCNT* gene, had a causal effect on the serum ALT level in DNAm.

**Table 3:** Causal Inference Analysis Results for the Top 35 CpG Sites

Chromosome	Position	Gene	Methylation to ALT					ALT to methylation				
			*β*_self change_	*p*_self change_	*β*_co-twin change_	*p*_co-twin change_	AVR	*β*_self change_	*p*_self change_	*β*_co-twin change_	*p*_co-twin change_	AVR
chr5	180,046,227	*FLT4*	− 0.325	0.117	− 1.171	0.006	3.599	3.52E-04	0.956	0.042	0.128	–
chr5	180,046,224	*FLT4*	− 0.327	0.112	− 1.166	0.006	3.571	4.12E-04	0.948	0.042	0.128	–
chr5	180,046,235	*FLT4*	− 0.324	0.123	− 1.174	0.006	3.629	3.15E-04	0.960	0.042	0.125	–
chr5	180,046,240	*FLT4*	− 0.323	0.128	− 1.178	0.006	3.651	2.68E-04	0.966	0.042	0.124	–
chr5	180,046,244	*FLT4*	− 0.321	0.132	− 1.182	0.007	3.681	1.98E-04	0.975	0.042	0.123	–
chr5	180,046,249	*FLT4*	− 0.319	0.139	− 1.189	0.007	3.729	8.33E-05	0.989	0.042	0.123	–
chr5	180,046,258	*FLT4*	− 0.311	0.154	− 1.195	0.008	3.843	− 2.24E-04	0.972	0.041	0.130	–
chr5	180,046,270	*FLT4*	− 0.304	0.164	− 1.210	0.008	3.981	− 0.001	0.917	0.041	0.143	–
chr5	180,046,275	*FLT4*	− 0.301	0.168	− 1.218	0.008	4.043	− 0.001	0.896	0.041	0.148	–
chr5	180,046,290	*FLT4*	− 0.299	0.153	− 1.228	0.005	4.109	− 0.001	0.850	0.040	0.160	–
chr5	180,046,305	*FLT4*	− 0.291	0.136	− 1.193	0.003	4.104	− 0.002	0.829	0.040	0.172	–
chr10	1,517,277	*ADARB2*	− 0.302	0.468	− 1.095	0.113	–	0.004	0.588	0.051	0.083	–
chr10	1,517,258	*ADARB2*	− 0.303	0.401	− 1.084	0.074	–	0.003	0.652	0.050	0.092	–
chr13	20,139,121	*MRPS31P2*	− 0.552	< 0.001	− 1.727	< 0.001	3.128	− 0.002	0.723	0.037	0.179	–
chr10	1,517,242	*ADARB2*	− 0.318	0.293	− 1.097	0.034	3.446	0.003	0.702	0.050	0.094	–
chr10	1,517,237	*ADARB2*	− 0.322	0.254	− 1.105	0.025	3.434	0.002	0.742	0.050	0.095	–
chr10	1,517,307	*ADARB2*	− 0.341	0.276	− 0.992	0.022	2.905	0.007	0.346	0.050	0.083	–
chr19	45,532,179	*RELB*	0.387	0.149	1.853	0.007	4.783	0.002	0.684	− 0.034	0.115	–
chr10	1,517,310	*ADARB2*	− 0.354	0.218	− 0.988	0.012	2.791	0.007	0.332	0.049	0.086	–
chr10	1,517,225	*ADARB2*	− 0.337	0.140	− 1.116	0.010	3.307	0.002	0.827	0.050	0.095	–
chr10	1,517,219	*ADARB2*	− 0.365	0.104	− 1.179	0.004	3.229	0.001	0.850	0.051	0.091	–
chr4	49,163,502	–	0.381	0.689	1.524	0.498	–	0.001	0.871	− 0.030	0.267	–
chr15	72,490,479	–	0.809	0.069	2.642	0.002	3.266	0.011	0.112	− 0.048	0.089	–
chr4	49,163,509	–	0.349	0.744	1.472	0.554	–	0.001	0.876	− 0.029	0.285	–
chr19	45,532,170	*RELB*	0.425	0.100	1.755	0.010	4.124	0.002	0.758	− 0.032	0.141	–
chr4	49,163,519	–	0.316	0.788	1.385	0.607	–	0.001	0.898	− 0.028	0.306	–
chr19	45,532,208	*RELB*	0.232	0.345	1.456	0.025	6.290	0.003	0.680	− 0.036	0.123	–
chr19	45,532,201	*RELB*	0.252	0.259	1.537	0.011	6.102	0.004	0.626	− 0.037	0.113	–
chr19	45,532,214	*RELB*	0.226	0.362	1.447	0.027	6.400	0.003	0.690	− 0.036	0.125	–
chr19	45,532,196	*RELB*	0.252	0.250	1.543	0.009	6.122	0.004	0.611	− 0.037	0.115	–
chr1	2,928,715	–	0.550	0.214	1.628	0.025	2.960	− 0.001	0.806	− 0.039	0.150	–
chr19	45,532,235	*RELB*	0.195	0.449	1.366	0.029	6.998	0.003	0.733	− 0.035	0.130	–
chr10	1,517,313	*ADARB2*	− 0.376	0.132	− 0.986	0.003	2.619	0.007	0.310	0.049	0.091	–
chr1	2,928,748	–	0.603	0.066	1.744	< 0.001	2.890	− 9.21E-05	0.987	− 0.040	0.153	–
chr1	2,928,733	–	0.581	0.134	1.711	0.004	2.947	− 4.83E-04	0.929	− 0.040	0.152	–

AVR: Absolute value of ratio, AVR =|*β*_co-twin change_| / |*β*_self change_|, AVR > 1.5 indicates a causal effect

### Region-based analysis

Fifty-two DMRs were found to be associated with serum ALT levels. Table 4 provides information on a portion of significant DMRs, while the details for all 52 DMRs are included in Table S3.

**Table 4:** Summary of Partial Significant Differentially Methylated Regions (DMRs) Associated with Serum ALT Levels

Chromosome	Start (bp)	End (bp)	Length	Stouffer–Liptak–Kechris (*slk*) corrected *p*-value	Gene	Nearest gene
chr5	180,045,726	180,046,393	28	1.53E-11	*FLT4*	
chr10	1,517,152	1,517,314	10	1.73E-10	*ADARB2*	
chr4	49,163,427	49,163,595	11	5.50E-09	–	*ENSG00000222437*
chr1	23,280,002	23,280,168	21	8.93E-08	*LACTBL1*	
chr19	45,531,921	45,532,286	15	1.72E-07	*RELB*	
chr16	32,289,939	32,290,060	14	1.79E-07	–	*ENSG00000259822*
chr10	43,428,611	43,429,087	20	7.57E-07	–	*ENSG00000229630*
chr2	130,975,639	130,975,784	12	2.66E-06	–	*RHOQP3*
chr2	186,794,503	186,794,653	12	7.97E-05	–	*RPL21P32*
chr19	14,584,404	14,584,550	18	1.59E-04	*PTGER1*	
chr1	2,491,130	2,491,396	13	1.73E-04	*TNFRSF14*	
chr17	46,645,892	46,646,045	10	3.50E-04	*HOXB3, HOXB-AS3*	

**Fig. 3:**
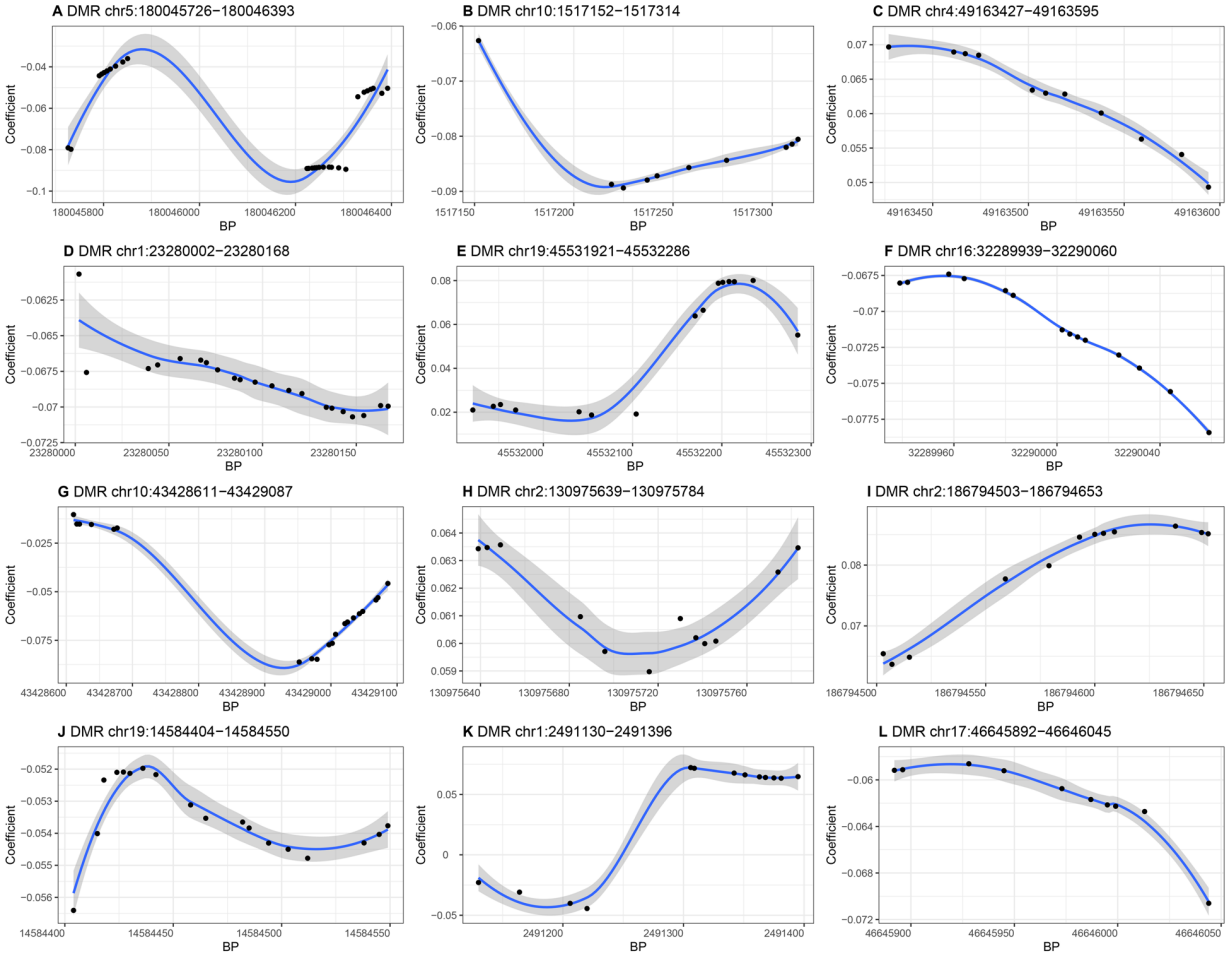
Potential mechanisms by which *FLT4* and *RELB* genes influence serum ALT levels

As shown in Fig. 3, some DMRs presented varying correlations with the serum ALT level. For example, Fig. 3A shows a DMR located in the *FLT4* gene, which is negatively correlated with serum ALT. Conversely, Fig. 3E depicts a DMR located in the *RELB* gene, which is positively correlated with the serum ALT level.

Among the 52 identified DMRs, 24 exhibited positive correlations with serum ALT levels, another 21 showed negative correlations, and the association trend for the remaining 7 DMRs remained uncertain. The complete set of 52 DMRs is displayed in the supplementary materials (Fig. S1).

### Ontology enrichment analysis

A total of 16,109 genomic cis-regulatory regions associated with various genes were identified from the EWAS results of serum ALT according to GRCh37/hg19 (Fig. S2a). Figs. S2b and c display the absolute distance and orientation of the genomic cis-regulatory areas with respect to the transcription start site (TSS).

Multiple pathways linked to serum ALT levels were found in the pathway and biological process enrichment studies, including low voltage-gated calcium channel activity and focal adhesion. Partial results are presented in Table 5, while all results with Bonferroni correction *p* < 0.05 are detailed in Table S4.

**Table 5:** Partial pathways and biological processes enriched for serum ALT levels

Ontology database	Term name	*p* value	Bonferroni correction *p* value	Fold enrichment	Observed region hits	Region set coverage
GO molecular function	low voltage-gated calcium channel activity	2.54E-99	9.37E-96	29.084	93	0.006
GO molecular function	DNA binding	3.27E-87	1.21E-83	1.304	4601	0.283
GO biological process	gene expression	9.30E-81	9.71E-77	1.242	5726	0.352
GO biological process	RNA metabolic process	2.71E-80	2.83E-76	1.251	5462	0.336
GO molecular function	thromboxane A2 receptor activity	1.19E-78	4.38E-75	149.335	44	0.003
GO biological process	thromboxane A2 signaling pathway	1.19E-78	1.24E-74	149.335	44	0.003
GO biological process	RNA biosynthetic process	2.64E-78	2.75E-74	1.279	4715	0.290
GO biological process	cortisol biosynthetic process	2.59E-77	2.70E-73	38.055	65	0.004
GO biological process	aldosterone biosynthetic process	2.59E-77	2.70E-73	38.055	65	0.004
GO biological process	transcription, DNA-dependent	5.02E-77	5.24E-73	1.279	4645	0.286
GO biological process	mitral valve formation	4.44E-75	4.64E-71	36.577	64	0.004
GO biological process	nucleic acid metabolic process	5.21E-72	5.44E-68	1.215	6053	0.372
Human phenotype	Genu recurvatum	9.34E-72	5.74E-68	7.240	143	0.009
Human phenotype	Difficulty climbing stairs	3.01E-71	1.85E-67	6.863	148	0.009
GO biological process	organic cyclic compound biosynthetic process	8.91E-70	9.30E-66	1.239	5222	0.321
GO molecular function	nucleic acid binding	1.89E-68	6.96E-65	1.222	5642	0.347
GO biological process	nucleobase-containing compound biosynthetic process	2.07E-67	2.16E-63	1.245	4951	0.305
GO biological process	macromolecule metabolic process	1.89E-66	1.98E-62	1.127	9649	0.594
GO biological process	macromolecule biosynthetic process	8.57E-66	8.95E-62	1.211	5815	0.358
GO biological process	cellular metabolic process	2.75E-65	2.87E-61	1.108	10,806	0.665
GO biological process	cellular nitrogen compound biosynthetic process	1.09E-64	1.14E-60	1.234	5076	0.312
Human phenotype	Urethral stricture	4.80E-64	2.95E-60	74.524	43	0.003
Human phenotype	Skin fragility with non-scarring blistering	4.80E-64	2.95E-60	74.524	43	0.003
Human phenotype	Onychogryposis of toenails	4.80E-64	2.95E-60	74.524	43	0.003
GO biological process	aromatic compound biosynthetic process	6.21E-64	6.48E-60	1.234	5036	0.310
GO biological process	regulation of macromolecule biosynthetic process	2.36E-63	2.47E-59	1.191	6357	0.391
GO biological process	metabolic process	2.65E-63	2.77E-59	1.095	11,579	0.713
GO molecular function	sequence-specific DNA binding	2.97E-62	1.10E-58	1.429	2171	0.134
GO biological process	regulation of RNA biosynthetic process	3.17E-62	3.31E-58	1.200	5942	0.366
GO biological process	cellular macromolecule biosynthetic process	4.78E-62	4.99E-58	1.208	5678	0.349
BioCyc pathway	alanine biosynthesis II	1.43E-10	4.76E-08	14.046	12	0.001
BioCyc pathway	alanine degradation	1.43E-10	4.76E-08	14.046	12	0.001
MSigDB pathway	Focal adhesion	3.44E-05	4.55E-02	1.210	456	0.028

### Results of validation

We selected candidate genes in accordance with the criteria provided in the ‘Methods’ section. Ultimately, we quantified 9 CpGs in the *FLT4* gene (originally 11, but 2 were not detected) and 7 CpGs in the *RELB* gene via the Sequenom MassARRAY platform. The validation cohort consisted of 216 individuals, comprising 54 cases and 162 controls. The baseline characteristics of the validation cohort are summarized as follows: In the ALT normal group, 102 participants were women, accounting for 63% of the group, with a median age of 49.5 years (IQR: 46–55). Similarly, in the ALT abnormal group, 34 participants were women (63%), with a median age of 49.5 years (IQR: 47–57).

There is statistically significant evidence (*p* < 0.05) of a substantial negative connection between serum ALT and four of the top CpGs in the *FLT4* gene. Furthermore, a significant positive relationship (*p* < 0.05) between the serum ALT level and three of the top CpGs in the *RELB* gene was confirmed. The results are presented in Supplementary Table S5.

## Discussion

To our knowledge, no previous methylation analyses of serum ALT have focused on the Chinese or East Asian population, highlighting a significant gap in the literature. This study investigated the epigenetic factors influencing serum ALT levels in the Chinese population by conducting an EWAS on a cohort of monozygotic twins. Our analysis revealed that 85 CpG sites were significantly associated with serum ALT levels. Additionally, we identified multiple genes, DMRs, and pathways that may elucidate the mechanisms underlying changes in the serum ALT level. For further validation, several candidate CpGs located in the *FLT4* and *RELB* genes were quantified and confirmed. The insights garnered from our analyses offer significant contributions to understanding the epigenetic regulation of liver function, and provides a theoretical basis for future investigations into whether the methylation status of these genes could serve as early diagnostic biomarkers for liver dysfunction.

While previous studies have focused on European cohorts [21], genetic background and environmental exposures differ across populations, potentially influencing DNA methylation patterns. Our study contributes novel insights into the epigenetic regulation of ALT levels in an East Asian population, an area that has been underexplored. Future studies in diverse populations are warranted to determine whether the identified epigenetic associations with ALT are generalizable across ethnic groups through EWAS or DNA methylation score [43], which can further expand epigenetic studies to other populations to gain a more comprehensive understanding of liver function regulation.

Among the CpG sites that ranked at the top of Table 2, the top 11 were located within the *FLT4* gene. The *FLT4* gene encodes VEGFR3, known for its involvement in the vascular endothelial growth factor (VEGF) and vascular endothelial growth factor receptor (VEGFR) signaling pathways [44], which is crucial for lymphangiogenesis, vascular remodeling, and hepatic microcirculation homeostasis [45], emerged as a critical locus in our study. Several animal studies have suggested mechanisms through which *FLT4* may influence ALT levels. One study suggested that VEGF might stimulate sinusoidal endothelial cells to produce cytokines such as hepatocyte growth factor. This process increases the expression of *Bcl-xL* in hepatocytes, thereby reducing apoptosis. Researchers have therefore postulated that via intercellular contacts, VEGF exerts a strong antiapoptotic effect on hepatocytes [46].

In addition, *FLT4* is involved in the focal adhesion pathway, a crucial regulator of cell adhesion, migration, and tissue remodeling. Our GREAT binomial test results (Table 5) indicated significant enrichment of this pathway, suggesting its potential link to serum ALT levels. The *FLT4* gene participates in the generation of focal adhesion kinase (FAK), which has been implicated in hepatic injury response and fibrosis progression [47, 48]. Moreover, studies have shown that VEGF interacts with nuclear factor erythroid 2-related factor 2 (Nrf2), inducing the Nrf2 signaling pathway to counteract oxidative stress. Through a positive feedback loop, VEGF further enhances Nrf2 activation and promotes its own expression, thereby protecting cells from oxidative damage [49]. This mechanism may contribute to hepatocyte protection against oxidative stress-induced injury, ultimately influencing serum ALT levels. These findings suggest that *FLT4* may contribute to liver function regulation through VEGF, FAK signaling, and Nrf2. However, further studies are needed to clarify whether *FLT4* methylation directly modulates these pathways in hepatocytes or sinusoidal endothelial cells.

**Fig. 4:**
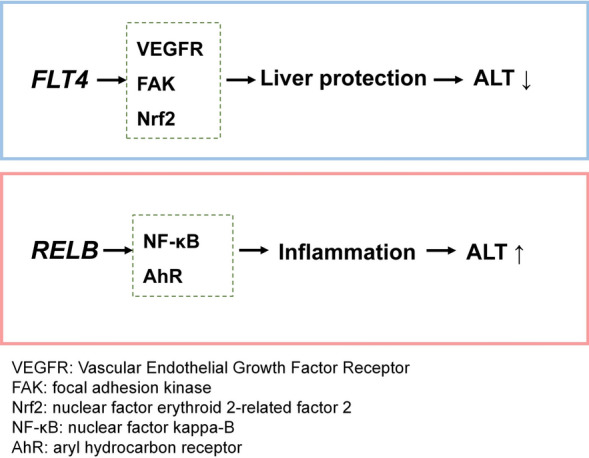
Schematic illustration of the potential mechanisms by which FLT4 and RELB influence serum ALT levels

Among the top CpGs, seven sites are located on the *RELB* gene. *RELB* belongs to the family of mammalian nuclear factor kappa-B (NF-κB) [50]. Many biological processes, including the immunological response, inflammatory responses, cell proliferation and survival, and development, are influenced by NF-κB [51, 52]. Consequently, hepatocyte damage and subsequent changes in serum ALT levels may be caused by the NF-κB signaling pathway, which may be involved in the biological processes of liver inflammation. Additionally, studies have shown that the use of NF-κB inhibitors can suppress *RELB* expression, thereby alleviating liver injury [53]. Moreover, *RELB* may interact with the aryl hydrocarbon receptor (AhR) to regulate the transcription of chemokines, thereby contributing to the inflammatory response [54]. These findings further suggest that *RELB* might influence serum ALT levels by impacting liver damage. To better illustrate the potential mechanisms through which *FLT4* and *RELB* might influence ALT levels, Fig. 4 provides a visual summary of the key pathways involved.

Some CpG sites may exhibit co-methylation patterns within specific genomic regions. To account for such regional effects, we performed DMR analysis, which confirmed significant associations between DNAm and serum ALT levels across different genomic regions where the top CpGs are located, including the sites in *FLT4* and *RELB*. While DMRs help capture biologically relevant patterns, the dynamic nature of DNA methylation differs from the stable linkage disequilibrium (LD) structures seen in GWAS. Additionally, the absence of a standardized LD-equivalent reference for methylation makes fine mapping in EWAS particularly challenging. Future studies incorporating large-scale methylation datasets and co-methylation network analyses may provide further insights into these regional methylation effects.

The results of causal inference consistently supported the directional effect of the majority of top CpGs on ALT levels. Furthermore, validation via the Sequenom MassARRAY platform in an independent cohort confirmed our initial findings, with significant correlations observed for CpGs in *FLT4* and *RELB*, which was consistent with the direction of the initial analysis. These additional layers of validation affirm the robustness and reliability of our conclusions.

The solitary CpG site (chr21, position: 47,808,888) in the *PCNT* gene showing a reverse causal effect, where ALT levels influence DNAm, is intriguing and warrants further investigation.

Using gene set enrichment analysis, significant gene sets associated with reduced voltage-gated calcium channel activity were found in genes linked to CpGs with low *p* values. Although direct research is limited, several studies have shown that calcium channel blockers can protect the liver. For example, a randomized controlled experiment showed that acute alcoholic hepatitis might be treated with amlodipine, a calcium channel antagonist [55]. Furthermore, R.J. Nauta et al. reported that in a model of ischemia‒reperfusion-induced liver injury, significant mitochondrial calcium loading and an increase in calcium and oxygen-derived free radicals during ischemia might cause mitochondrial damage in a model of ischemia‒reperfusion-induced liver injury [56]. These studies suggest that calcium channel activation may be associated with liver injury, subsequently affecting serum ALT levels.

This study has several noteworthy advantages. First, MZ twins share identical genetic material, which significantly reduces genetic confounding when studying epigenetic modifications [27]. This unique feature allows for a more accurate assessment of environmental influences on DNA methylation and serum ALT levels without the interference of genetic variability. Second, by performing causal inference analysis and validating the findings in an independent cohort, we were able to confirm that DNAm changes at most of the top CpGs have a directional effect on ALT levels. This combination of causal inference and validation strengthens the evidence for the potential causative role of these epigenetic modifications. Another key strength of our study is the incorporation of an independent community-based validation cohort, which enhances the robustness and potential generalizability of our findings. By applying PSM, we minimized confounding effects related to age and sex, strengthening the validity of our DNAm-ALT associations. These strengths collectively highlight the robustness and applicability of our findings for understanding the epigenetic regulation of liver function.

Although our study offers valuable insights, it has several limitations. The recruitment and identification of eligible twins presented considerable challenges, resulting in a relatively restricted sample size. While the trait-discordant twin design enhances statistical power compared to conventional cross-sectional or case‒control studies, the relatively small sample size may still limit the ability to detect subtle DNAm-ALT associations. Additionally, despite our previous research demonstrating a high heritability of serum ALT levels (65%) [11] and an estimated statistical power of approximately 80% with over 60 twin pairs [26], a larger sample size could further strengthen the robustness of our findings. Moreover, as our study population was limited to Chinese individuals, the generalizability of our results to other ethnic groups remains uncertain. Future studies with larger and more diverse populations are needed to validate these associations and explore the underlying biological mechanisms. In addition, while our study focused on ALT as a key marker of liver function, future research could benefit from a broader exploration of liver enzymes such as aspartate aminotransferase (AST) and gamma-glutamyl transferase (GGT) to further elucidate the epigenetic mechanisms underlying liver health. As part of our ongoing research, we plan to expand our investigation to include these biomarkers, which may provide additional insights into the epigenetic regulation of liver function. Finally, although we performed validation in a community-based population, further validation in larger independent cohorts or through functional experiments would strengthen our findings.

## Conclusion

In conclusion, our work establishes a basis for further investigation into the epigenetic processes underlying liver health and highlights the critical role that DNAm plays in controlling serum ALT levels. The identification of specific CpGs in *FLT4* and *RELB* associated with ALT levels offers promising avenues for developing epigenetic biomarkers and therapeutic interventions for liver injury. The robustness of our findings, supported by DMR analysis, causal inference, and external validation, underscores the potential of DNAm as a key player in the regulation of liver function.

## Supplementary Information

Additional file 1.

Additional file 2.

## Data Availability

Data supporting the findings of this study are available from the corresponding author upon reasonable request.

## Abbreviations

ALT: Alanine aminotransferase
DMRs: Differentially methylated regions
DNAm: DNA methylation
EWAS: Epigenome-wide association study
GO: Gene ontology
GREAT: Genomic region enrichment of annotations tool
ICE FALCON: Inference about causality through examination of the familial confounding method
KEGG: Kyoto encyclopedia of genes and genomes
LD: Linkage disequilibrium
MZ: Monozygotic
PSM: Propensity score matching
RRBS: Reduced representation bisulfite sequencing
