# Supplementary material for: DNA methylation associated with the serum alanine aminotransferase concentration: evidence from Chinese monozygotic twins
Source: Clin Epigenetics. 2025 Apr 28;17:65. doi: 10.1186/s13148-025-01869-1 (PMC12039056; doi:10.1186/s13148-025-01869-1)
Supplement: Supplementary file 1 — Additional file 1. [file 13148_2025_1869_MOESM1_ESM.docx]

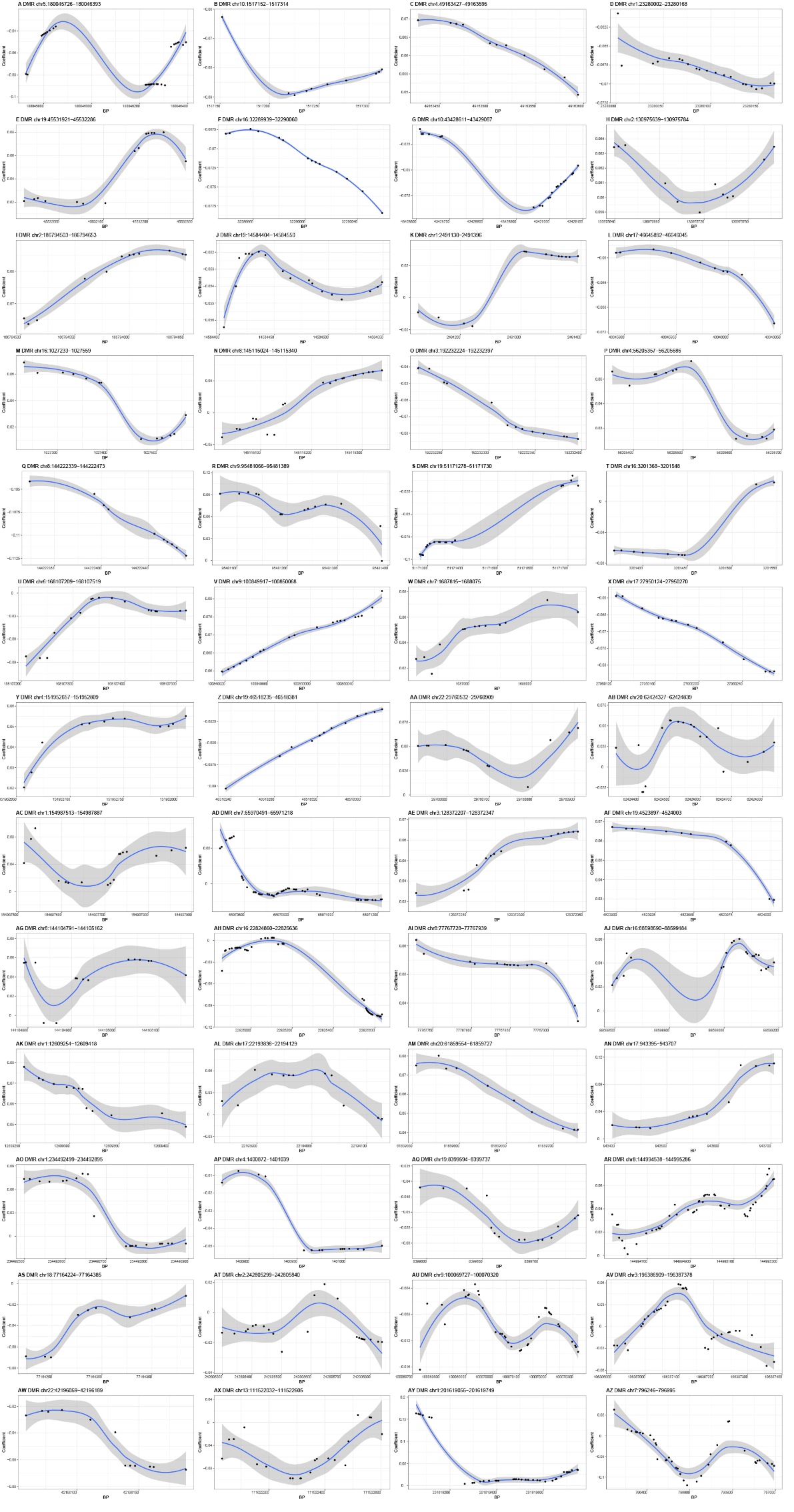


Figure S1 Complete Set of 52 Differentially Methylated Regions (DMRs) Associated with Serum ALT Levels


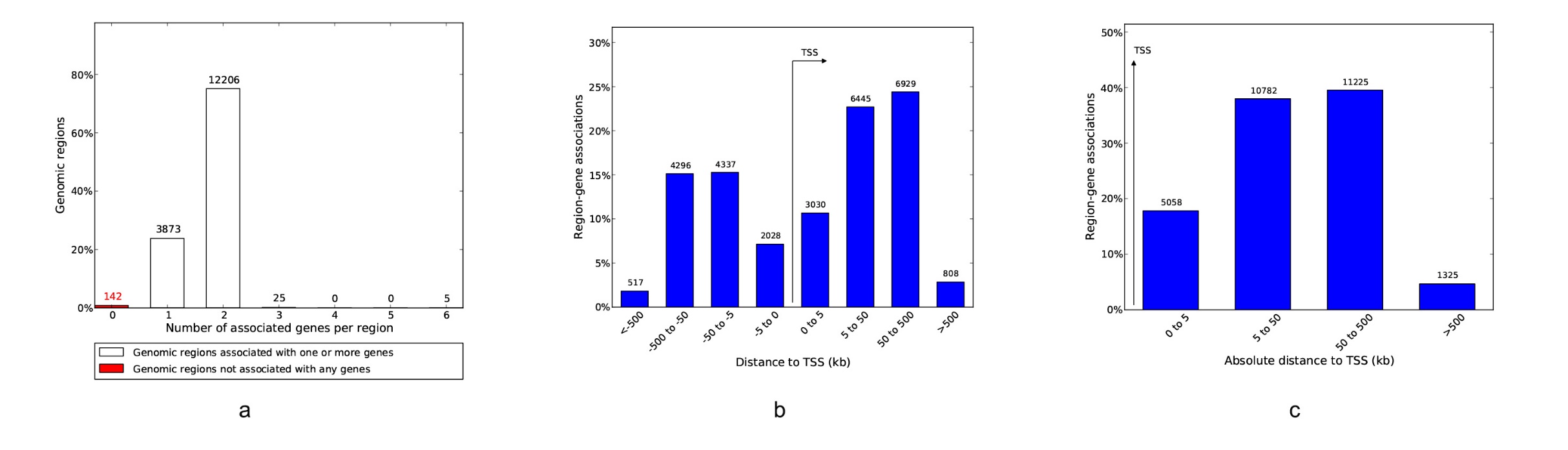


Figure S2 Genomic Cis-Regulatory Regions Identified in EWAS of Serum ALT Levels. a. Distribution of Genomic Cis-Regulatory Regions Related to Serum ALT Levels (GRCh37/hg19); b. Orientation of Genomic Cis-Regulatory Regions with Respect to Transcription Start Site (TSS); c. Absolute Distance of Genomic Cis-Regulatory Regions from Transcription Start Site (TSS)
